# Fabrication of Cr-ZnFe_2_O_4_/S-g-C_3_N_4_ Heterojunction Enriched Charge Separation for Sunlight Responsive Photocatalytic Performance and Antibacterial Study

**DOI:** 10.3390/molecules27196330

**Published:** 2022-09-26

**Authors:** Ping Zhang, Tehreem Munawar, Raya Soltane, Mohsin Javed, Guocong Liu, Shahid Iqbal, Muhammad Azam Qamar, Ayed A. Dera, Hamad Alrbyawi, M. Alfakeer, Sameh Rabea, Eslam B. Elkaeed

**Affiliations:** 1 School of Chemistry and Materials Engineering, Huizhou University, Huizhou 516007, China; 2 Department of Chemistry, School of Science, University of Management and Technology, Lahore 54770, Pakistan; 3 Department of Basic Sciences, Adham University College, Umm Al-Qura University, Makkah 21955, Saudi Arabia; 4 Department of Biology, Faculty of Sciences, Tunis El Manar University, Tunis 1068, Tunisia; 5 Department of Clinical Laboratory Sciences, College of Applied Medical Sciences, King Khalid University, Abha 61421, Saudi Arabia; 6 Pharmaceutics and Pharmaceutical Technology Department, College of Pharmacy, Taibah University, Medina 42353, Saudi Arabia; 7 Department of Chemistry, College of Science, Princess Nourah bint Abdulrahman University, P.O. Box 84428, Riyadh 11671, Saudi Arabia; 8 Department of Pharmaceutical Sciences, College of Pharmacy, AlMaarefa University, Riyadh 13713, Saudi Arabia

**Keywords:** photocatalyst, hydrothermal method, S-g-C_3_N_4_, nanocomposite, polluted water

## Abstract

There has been a lot of interest in the manufacture of stable, high-efficiency photocatalysts. In this study, initially Cr doped ZnFe_2_O_4_ nanoparticles (NPs) were made via surfactant-assisted hydrothermal technique. Then Cr-ZnFe_2_O_4_ NPs were modified by incorporating S-g-C_3_N_4_ to enhance their photocatalytic efficiency. The morphological, structural, and bonding aspects were analyzed by XRD, FTIR, and SEM techniques. The photocatalytic efficiency of the functional Cr-ZnFe_2_O_4_/S-g-C_3_N_4_ (ZFG) heterostructure photocatalysts was examined against MB under sunlight. The produced ZFG-50 composite has the best photocatalytic performance, which is 2.4 and 3.5 times better than that of ZnFe_2_O_4_ and S-g-C_3_N_4_, respectively. Experiments revealed that the enhanced photocatalytic activity of the ZFG nanocomposite was caused by a more effective transfer and separation of photo-induced charges. The ZFG photocatalyst can use sunlight for treating polluted water, and the proposed modification of ZnFe_2_O_4_ using Cr and S-g-C_3_N_4_ is efficient, affordable, and environmentally benign. Under visible light, Gram-positive and Gram-negative bacteria were employed to ZFG-50 NCs’ antimicrobial activity. These ZFG-50 NCs also exhibit excellent antibacterial potential.

## 1. Introduction

Pollution is one of the most serious dangers that humans face. Pollution of various aquatic ecosystems is the most widespread type that has a significant impact on living things. Industrial dyes are known to be dangerous to people, especially when soluble in water [1]. Methylene blue (MB) dye is one of the most popular dyes that pollute aquatic habitats. Therefore, developing a good method for the cleanup of wastewater is critical. To remove organic dyes from wastewater, many methods have been used, including biodegradation, adsorption, filtering, sedimentation, and coagulation [2,3]. However, these procedures did not produce good results in terms of dye degradation. Scientists have demonstrated that photocatalytic decomposition is an appropriate alternative technique for the enhanced decomposition of numerous contaminants due to its high efficiency and low cost. Moreover, endorsing photocatalysis does not necessitate the use of other methods to remove the byproducts [1,4,5,6]. 

The g-C_3_N_4_ semiconductor has shown significant photocatalytic proficiency under visible light, as a result of its favorable characteristics such as high stability and a reduced band gap energy, which improves its capacity to absorb visible radiations [7,8,9,10]. However, the quick recombination of photoinduced e^−^/h^+^ pairs in the g-C_3_N_4_ makes it unsuitable for use as a photocatalyst [11,12,13]. As a result, numerous attempts to remove this limitation have been made, including vacancy, heterojunction formation, and mixing the g-C_3_N_4_ with some other metal oxide and nonmetals such as S [14,15]. By stacking its 2p orbitals on the VB of bulk g-C_3_N_4_, S-doping alters the bandgap of g-C_3_N_4_ and enhances the mobility and separation of the e-h pairs. Hong et al. reported that the photocatalytic H_2_ production efficiency of mesoporous S-g-C_3_N_4_ is 30 times more than pure g-C_3_N_4_ [16]. Similarly, S-g-C_3_N_4_ had an approximately 1.38 times greater photocatalytic CO_2_ reduction rate than pure g-C_3_N_4_ [14]. Under visible light, porous S-g-C_3_N_4_ had better adsorption and photocatalytic degradation of Rhodamine B dye than pure g-C_3_N_4_ [17]. 

S-doping has been shown to change the structural properties of g-C_3_N_4_, reduce its Eg value, and enhance the e^−^/h^+^ pair separation efficiency both theoretically and empirically [18]. The heterogeneous photocatalyst’s nanosheet structure, on the other hand, provides a large number of active sites for the reaction, along with increased surface area and reduced recombination between photoinduced charges. The separation efficiency of photo-produced charges on the g-C_3_N_4_ can be expanded by combining it with another good semiconductor like ZnFe_2_O_4_, and the resulting heterojunction can be used for wastewater treatment [19,20,21,22].

Zinc ferrite is a spinel ferrite with all Fe^3+^ ions in the octahedral sites and Zn^2+^ ions in the tetrahedral sites. Because of its unusual catalytic and magnetic capabilities, it is a promising material. Many studies have shown that doping ZnFe_2_O_4_ with appropriate metal ions improves optical and photocatalytic characteristics [23,24]. Patil et al. used the co-precipitation approach to manufacture Gd^3+^ doped ZnFe_2_O_4_ nanoparticles, which demonstrated improved MB degradation of roughly 99% as compared to pure ZnFe_2_O_4_ (95% degradation in 240 min) [25]. According to Ajithkumar et al., yttrium-doped zinc ferrite made by solution combustion technique showed 95% MB degradation in 180 minutes [26]. Y-ZnFe_2_O_4_ has higher photocatalytic effectiveness than pure zinc ferrite. Under visible light, cobalt-doped zinc ferrite decomposed methylene blue more efficiently than ZnFe_2_O_4_. Many researchers have concluded that ZnFe_2_O_4_ has finite band gap energy and hence might form an effective heterojunction when combined with g-C_3_N_4_ [27].

Moreover, the advanced ZnFe_2_O_4_/g-C_3_N_4_ nanocomposite, which plays a role in increasing photocatalytic efficiency, may achieve longer separation between photoexcited charges [28]. Owing to the improved charge separation abilities, it is suggested to produce M-ZnFe_2_O_4_/S-g-C_3_N_4_ heterojunction to realize significant photocatalytic performance [29]. In this probe, hybrid ZFG-50 nanocomposites have been synthesized successfully via a surfactant (PEG) assisted hydrothermal process. The photocatalytic characteristics of synthesized materials were investigated using MB, an organic pollutant. In step one, the series of chromium-doped zinc ferrite (Cr-ZnFe_2_O_4_) nanoparticles were synthesized with varying chromium percentages (0.5, 1, 3, 5, 7, and 9 wt. %). The effect of Cr^3+^ substitution on photocatalytic properties of zinc ferrite was observed. The 7% Cr-ZnFe_2_O_4_ sample manifested the best absorption of solar light and degradation efficiency. In step two, the 7% Cr-ZnFe_2_O_4_ nanoparticles were homogenized with diverse concentrations of S-g-C_3_N_4_ (10, 30, 50, and 70 wt. %) to produce ZFG-50 with enhanced photocatalytic activity. The 7% Cr-ZnFe_2_O_4_/50% S-g-C_3_N_4_ nanocomposite executed the best photocatalytic activity as compared to pure ZnFe_2_O_4_, 7% Cr-ZnFe_2_O_4_, and S-g-C_3_N_4_. Results depicted that the enhanced photocatalytic activity of 7% Cr-ZnFe_2_O_4_/50% S-g-C_3_N_4_ nanocomposite was because of the enhanced absorption of sunlight and better separation of e^−^/h^+^ pairs between Cr-ZnFe_2_O_4_ and S-g-C_3_N_4_. To the best of our knowledge, the synthesis of ZFG-50 heterojunctions via the hydrothermal approach has never been used. The precursors used for the synthesis are low-cost, and the synthesized ZnFe_2_O_4_/S-g-C_3_N_4_ heterojunctions are not reported to be used as photocatalysts. The synthesized material may have potential applications in the field of water purification.

## 2. Experimental

### 2.1. Chemicals

Zinc Sulphate Heptahydrate (ZnSO_4_·7H_2_O), Iron (III) Chloride Anhydrous (FeCl_3_), Chromium (III) Chloride Hexahydrate (CrCl_3_·6H_2_O), Sodium Hydroxide (NaOH), Thiourea (CH_4_N_2_S), Polyethylene Glycol, and Methylene Blue (C_16_H_18_ClN_3_S) were purchased from Merck (Darmstadt, Germany) and used. 

### 2.2. Synthesis of Chromium Doped Zinc Ferrites

A surfactant-assisted hydrothermal technique was employed to fabricate, a set of chromium doped zinc ferrites (Cr-ZnFe_2_O_4_) with different chromium percentages (0.5, 1, 3, 5, 7, and 9 wt. %) [12]. For the preparation of 0.5% Cr-ZnFe_2_O_4_ three solutions A, B and C were made before synthesis. Solution A: 40 mL of deionized water were mixed with 0.0169 g of CrCl_3_·6H_2_O. Solution B: 40 mL of deionized water were used to emulsify 2.8624 g of ZnSO_4_.7H2O. Solution C: In 40 mL of deionized water, 3.244 g of FeCl_3_ was dissolved. Then, 10 mL of PEG-400 was added as a surfactant to the mixture of solutions A, B, and C in order to prevent the agglomeration of nanoparticles. The suspensions were then moved to a Teflon-lined autoclave after the pH of the resulting solution was adjusted to 11 by adding a 6 M NaOH solution. The autoclave was placed in a 175 °C oven for ten hours before being removed to cool to room temperature. The resulting precipitates were then filtered off and washed with deionized H_2_O and absolute ethanol and then, finally dried at 85 °C in an oven. The same process was applied to synthesize other percentages (0, 1, 3, 5, 7, and 9 wt. %) of Cr-ZnFe_2_O_4_. 

### 2.3. Synthesis of S-g-C_3_N_4_

S-g-C_3_N_4_ was produced via thermal polycondensation of thiourea to 570 °C for 5 h at 5 °C min^−1^ in a muffle furnace. It was then allowed to cool to ambient temperature and stored the resulting yellowish S-g-C_3_N_4_ [14].

### 2.4. Synthesis of Cr-ZnFe_2_O_4_/S-g-C_3_N_4_

**Figure 1:**
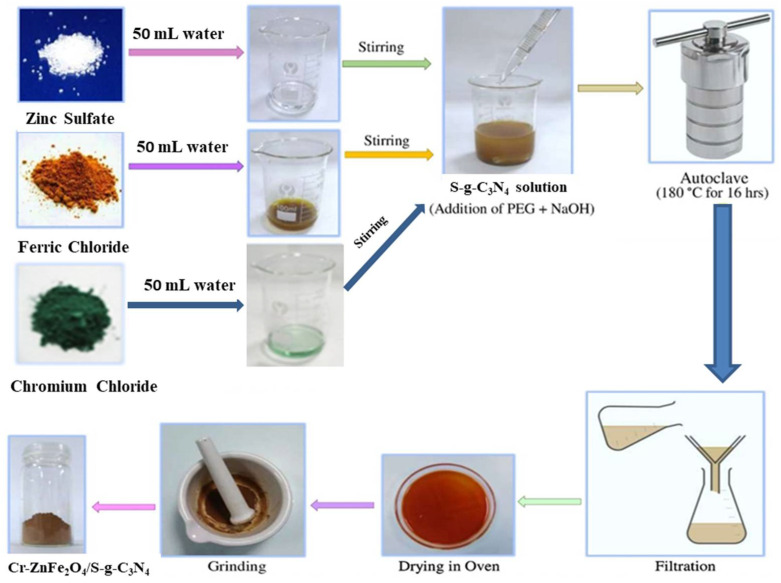
Schematic representation for the synthesis of Cr-ZnFe_2_O_4_/S-g-C_3_N_4_**.**

A range of ZFG-50 nanocomposites was made by incorporating 7% Cr-ZnFe_2_O_4_ with different concentrations of S-g-C_3_N_4_ (10, 30, 50, 60, and 70 wt. %) via surfactant-assisted hydrothermal process [30,31]. For the preparation of 7%Cr-ZnFe_2_O_4_/10%S-g-C_3_N_4_, four solutions A, B, C, and D were made before synthesis. Mixtures of 0.2346 g of CrCl_3_.6H_2_O in 30mL of water (Solution A), 2.6742 g of ZnSO_4_·7H_2_O in 30mL of water (Solution B), 3.244 g of FeCl_3_ in 30mL of water (Solution C), and 0.18 g of S-g-C_3_N_4_ in 30mL of water (Solution D) were dissolved in separate beakers and stirred. The solutions A, B, and C were added to solution D and homogenized for 45 minutes along with the addition of 10 mL of polyethylene glycol (PEG-400) as a surfactant. The next steps were the same as for the synthesis of Cr-ZnFe_2_O_4_ NPs. Moreover, the same process was repeated to synthesize the 7% ZFG-50 containing the (30, 50, 60, and 70 wt. %) of S-g-C_3_N_4_. The schematic diagram (Figure 1) depicts the synthesis procedure for ZFG-50 NCs, and Table 1 lists the precise composition.

### 2.5. Photocatalytic Activity

The photocatalyzed dye degradation activity of all synthesized photocatalysts was evaluated under the irradiation of solar light. The reference contaminant was an aqueous solution of the organic dye methylene blue (MB). A 100 mL solution of MB was diffused with 0.2 g of each photocatalyst (10 mg L^−1^). To achieve the adsorption-desorption equilibrium, the suspension was sonicated for 15 min, followed by 30 min of darkness. After that, the suspension was placed in an open space with sun light, and aliquots of 5 mL were taken every 30 min. After centrifugation, the sample’s photocatalytic activity was assessed using a UV-vis spectrophotometer.

## 3. Results and Discussion

### 3.1. XRD Analysis

**Figure 2:**
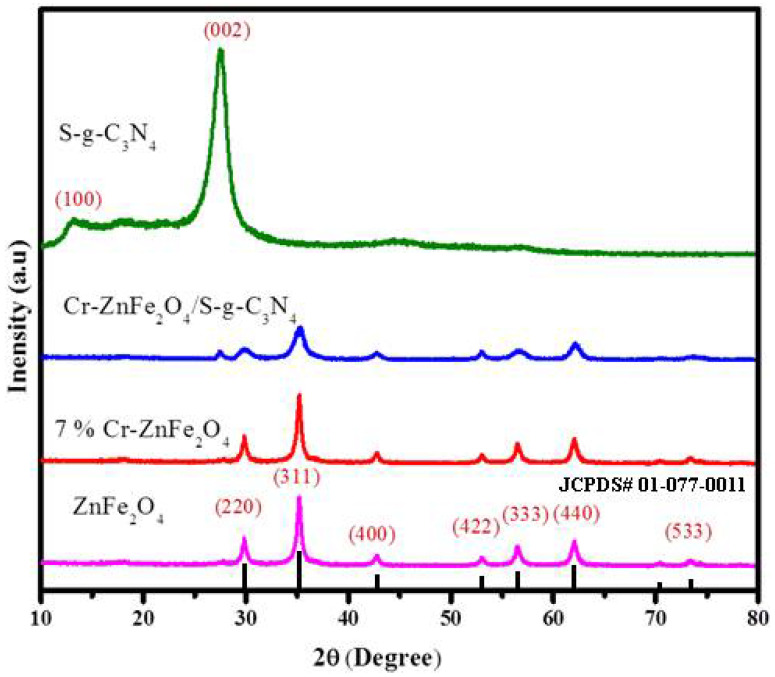
XRD spectrum of composites of ZnFe_2_O_4_, S-g-C_3_N_4_, 7% Cr-ZnFe_2_O_4_, 7% Cr-ZnFe_2_O_4_/S-g-C_3_N_4_.

Figure 2 shows the X-rays diffractogram of ZF, 7% Cr-ZnFe_2_O_4_, SG, and ZFG50 samples. Seven peaks were observed in the case of pure ZnFe_2_O_4_ with crystal facets (220), (311), (400), (422), (333), (440), and (533) at 2θ = 29.8°, 35.1°, 42.7°, 53°, 56.7°, 62.2°, and 73.8° that fitted well with the pattern of standard ZnFe_2_O_4_ with JCPDS file 01-077-0011 [32]. Two characteristic peaks were observed in the XRD pattern of SG, the crystal plane (002) was attributed to the interlayer assembling of aromatic systems and the plane (100) was ascribed to the inter-planar arrangement of aromatic systems [33,34]. After coupling with SG, the crystal phase of Cr-ZnFe_2_O_4_ stays unchanged, and the (002) crystal plane of the SG (weak) was indicated in the composite systems. Moreover, the XRD pattern shows no other impurity phase, indicating that ZFG50 is a two-phase nanocomposite. In 7%Cr-ZnFe_2_O_4_/50%S-g-C_3_N_4_ composites, owing to high crystallinity of Cr-ZnFe_2_O_4_ and low concentration of SG the characteristic peaks of Cr-ZnFe_2_O_4_ are prominent. Further, the crystal structure of Cr-ZnFe_2_O_4_ in the ZFG50 composite is unaffected by the addition of SG [35,36,37].

### 3.2. TEM, EDX, and XPS Analyses

**Figure 3:**
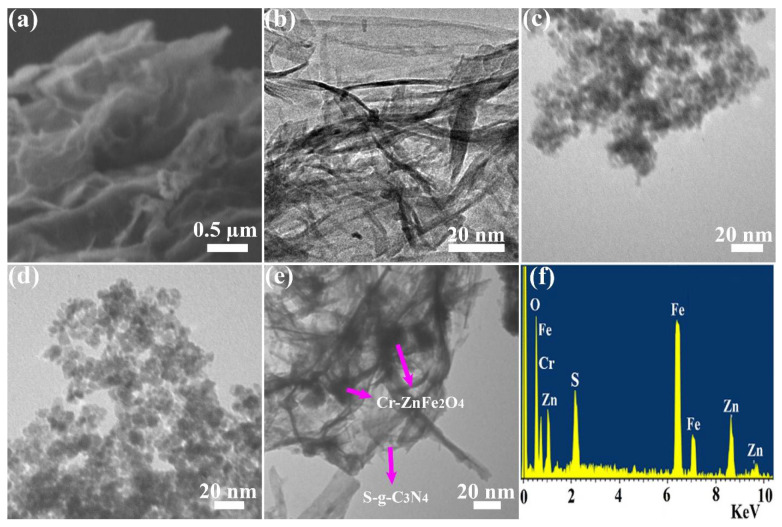
(**a**) SEM profile of S-g-C_3_N_4_, TEM profiles of (**b**) S-g-C_3_N_4_, (**c**) ZnFe_2_O_4_, (**d**) 7% Cr-ZnFe_2_O_4_, and (**e**) 7% Cr-ZnFe_2_O_4_/50S-g-C_3_N_4_ NCs. (**f**) EDX of 7% Cr-ZnFe_2_O_4_/50S-g-C_3_N_4_ NCs.

To evaluate the morphology of the synthesized photocatalysts, SEM and TEM micrographs were taken. The lamellar sheet-like structure is seen in the SEM and TEM pictures of pure S-g-C_3_N_4_ (Figure 3a,b). On the other hand, pure ZnFe_2_O_4_ and Cr-ZnFe_2_O_4_ that have been doped with Cr reveal very non-uniform spherical-like particles, as illustrated in Figure 3c,d, respectively. TEM was used to verify further how S-g-C_3_N_4_ and Cr-ZnFe_2_O_4_ nanoparticles interacted. The carbon nitride sheets were seen to be coated by the Cr-ZnFe_2_O_4_ nanoparticles in the TEM picture of the ZFG-50 NCs.

Figure 3e shows the TEM picture of the ZFG-50 NCs with a 7% metal oxide content. The S-g-C_3_N_4_ nanosheets’ surface has Cr-ZnFe_2_O_4_ nanoparticles deposited on it, as seen by the TEM pictures. The surface of the S-g-C_3_N_4_ nanosheets had evenly dispersed particles with an average size of 19 nm, according to the TEM pictures. By subjecting the composite to an ultrasonic treatment to prepare TEM samples, it was shown that the contact between the S-g-C_3_N_4_ sheet and nanoparticles is quite strong. When exposed to light, the S-g-C_3_N_4_ sheets and Cr-ZnFe_2_O_4_ particles seem to form a heterojunction, making it easier to boost the nanocomposite’s photocatalytic activity and separate the electron-hole in the opposite direction to produce the reactive species needed for dye mineralization. The EDX elemental mapping of the ZFG-50 NCs is also shown in Figure 3f, demonstrating that the principal elements of the ZFG-50 were Cr, Fe, Zn, O, C, and N. As shown in Figure S1, ZFG-50 was examined using XPS to ascertain its chemical composition and the electronic states of each of its constituent parts. Additionally, the XPS analysis supported the TEM and EDX findings that the Cr-ZnFe_2_O_4_/ S-g-C_3_N_4_ included ZnFe_2_O_4_, S-g-C_3_N_4_ and Cr.

### 3.3. FTIR Analysis

**Figure 4:**
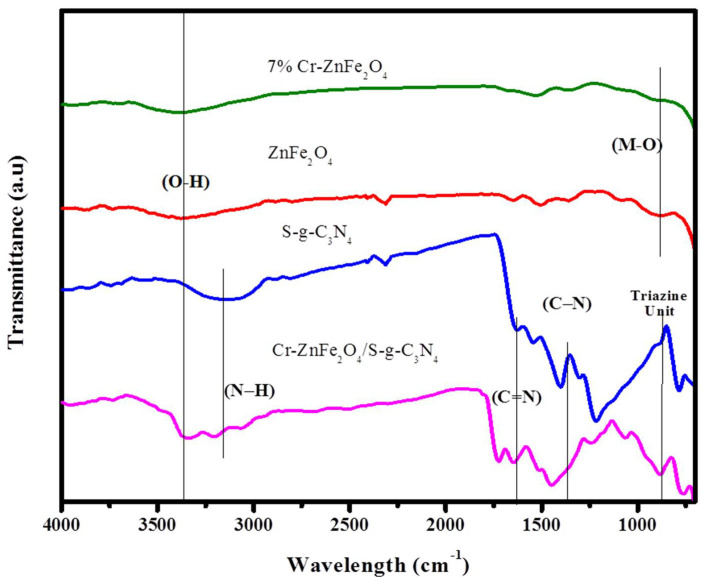
FTIR Spectrum of composites of ZnFe_2_O_4_, S-g-C_3_N_4_, 7% Cr-ZnFe_2_O_4_, 7% Cr-ZnFe_2_O_4_/S-g-C_3_N_4_.

The FTIR spectrum of ZF, 7% Cr-ZnFe_2_O_4_, SG and ZFG-50 samples is shown in Figure 4. The two active bands 3355 cm^−1^ and 834 cm^−1^ are observed in the FTIR spectra of zinc ferrite and 7% Cr-ZnFe_2_O_4_ [38]. These active bands are characteristic of the spinel structure of zinc ferrite nanoparticles. The band at 3355 cm^−1^ is attributed due to the stretching vibrations of the O-H bond of the free or absorbed water, whereas the band at 834 cm^−1^ is ascribed due to the stretching vibration of the Zn-O bond [39,40]. The band observed in composites at wavelength range 2800 cm^−1^ to 3400 cm^−1^ is attributed to N-H stretching, whereas a sharp peak observed at 870 cm^−1^ in all samples is due to the out-of-plane bending vibration of the tri-s-triazine ring of SG. The bands at 1600–1200 cm^−1^ were allocated to CN heterocycles (C=N and C-N) stretching vibrations, confirming the presence of S-g-C_3_N_4_ in composite samples [8,15]. Then, using the UV-vis spectra, the light-absorption of the designed photocatalysts ZnFe_2_O_4_, S-g-C_3_N_4_, and ZFG-50NCs was measured (Figure S2). The BET surface area was determined to be 9.23, 14.31, 27.11, and 63.78 m^2^/g for all formulations: ZnFe_2_O_4_, S-g-C_3_N_4_, and ZFG-50NCs (Figure S3).

### 3.4. Photocatalytic Degradation Study

**Figure 5:**
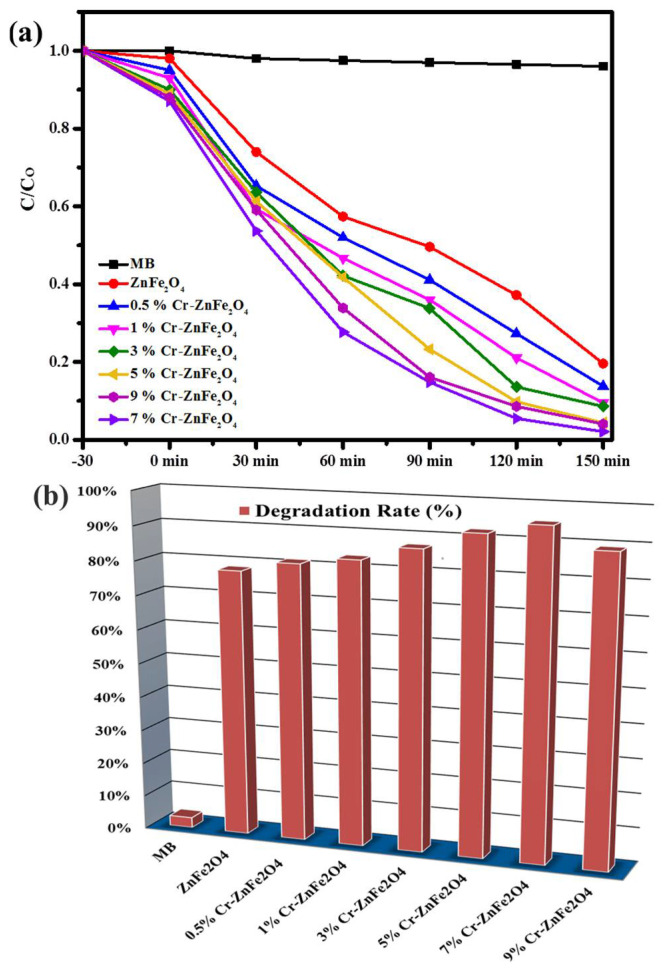
Photocatalytic activity of Cr-ZnFe_2_O_4_ NPs against MB (**a**) % Degradation of MB by Cr-ZnFe_2_O_4_ NPs (**b**).

**Figure 6:**
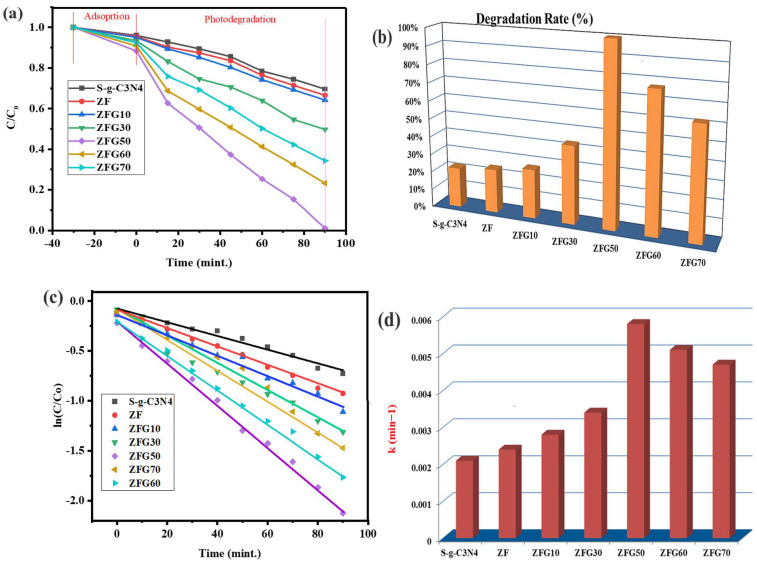
Photocatalytic degradation rate (**a**); % degradation (**b**); kinetic characteristics (**c**); and the rate constant (k) values of degradation of MB by ZFG NCs (**d**).

Under two phases, the photocatalytic activity of synthesized samples was investigated in the sunshine. The photocatalytic activities of ZnFe_2_O_4_ and Cr-ZnFe_2_O_4_ NPs (Figure 5a) were first investigated using an aqueous methylene blue solution in the presence of sunlight. A UV-vis spectrophotometer with a wavelength of 200–800 nm was used to track the dye degradation rate (Figure 5b). From the degradation contours (Figure S4) and % degradation plots (Figure 5b), the photocatalytic activity of chromium-doped zinc ferrite nanoparticles increased by increasing the Cr^+3^ doping up to 7 wt. %. Because the Cr^+3^ doping decreases the bandgap of ZnFe_2_O_4_, which facilitates the e^−^/h^+^ pair generation. 7% Cr^+3^ doping was the optimal concentration of Cr^+3^ ions. Increasing Cr^+3^ ions concentration beyond this (<7 wt. %.) leads to a decrease in photocatalytic activity of Cr-ZnFe_2_O_4_ NPs (Figure 6a,b). The observed degradation efficiencies of Cr-ZnFe_2_O_4_ catalysts with different chromium percentages (0, 0.5, 1, 3, 5, 7, and 9 wt. %) were 78%, 81%, 83%, 87%, 92%, 95%, and 89%, respectively, after 150 min of sunlight irradiation. Thus, the 7% Cr-ZnFe_2_O_4_ NPs exhibited the maximum photocatalytic efficiency as compared to other nanoparticles.

In the next step, the 7% Cr-ZnFe_2_O_4_ NPs were homogenized with diverse amounts of S-g-C_3_N_4_ (as given in Table 1) to develop ZFG-50(ZFG) NCs and their photocatalytic activity was checked after every 15 min interval. Before sunlight exposure, the fabricated NCs were placed in the dark to establish adsorption-desorption equilibrium between dye and the S-g-C_3_N_4_, ZF, ZFG10, ZFG30, ZFG50, ZFG60, and ZFG70 catalysts and the corresponding adsorbed amounts of MB are displayed in Figure 6c. The graph (Figure 6a) clearly shows that the samples absorbed relatively little amounts of dye. Then samples were exposed to sunlight and the ZFG-50 NCs exhibits maximum dye degradation as compared to other samples (Figure 6a). From the degradation contours (Figure S5) and % degradation plots (Figure 6b), it could be observed that on enhancing SG contents in the ZFG NCs, the dye degradation was increased up to ZFG50 NCs (containing 50% S-g-C_3_N_4_) and then decreased for ZFG60 and ZFG70 (<50% S-g-C_3_N_4_). The observed degradation efficiencies of SG, ZF, ZFG10, ZFG30, ZFG50, ZFG60, and ZFG70 catalysts were 23.47%, 26%, 31%, 51%, 100%, 70%, and 63.28%, respectively, after 90 min of sunlight irradiation. Improved charge separation and transfer via Cr-ZnFe_2_O_4_ and S-g-C_3_N_4_ coupling, as well as higher visible light absorption due to Cr doping in ZnFe_2_O_4_, may account for the improved degradation by ZFG [7,38,41]. Figure 6b depicts the % photocatalytic degradation of MB by NCs. The Langmuir–Hinshelwood model was applied to explain the kinetics [42]. It is evident that the dye degradation by the NCs under sunlight is fit to pseudo-first-order kinetics (Figure 6c). The rate constant (k) values are summarized in Table 2 and given in Figure 6d. 

ZFG50 (0.0058 min^−1^) and SG (0.0021 min^−1^) had the greatest and lowest “k” values, respectively. The ZFG50 NCs completely mineralized the MB in 90 min and its “k” value was 2.4 and 3.5 times more than that of SG and ZF respectively. As the S-g-C_3_N_4_ concentration increase from 10% to 50% in the ZFG NC, the dye degradation also enhances and then drops yonder this concentration (<50%). Thus, inherently, 50% S-g-C_3_N_4_ is the ideal concentration for the ZFG NC. Further increase in S-g-C_3_N_4_ concentration might produce e–h pair combination centers, which successively decrease the photocatalytic efficiency [43,44]. To further analyse this rationalization, a preliminary investigation is required. As shown in Table 3, the photocatalytic efficiency of ZFG50 NC is significantly higher than various prior reported research. Since the ZFG50 NC was found to be the most efficient photocatalyst and so it was further used in the recycling study.

**Figure 7:**
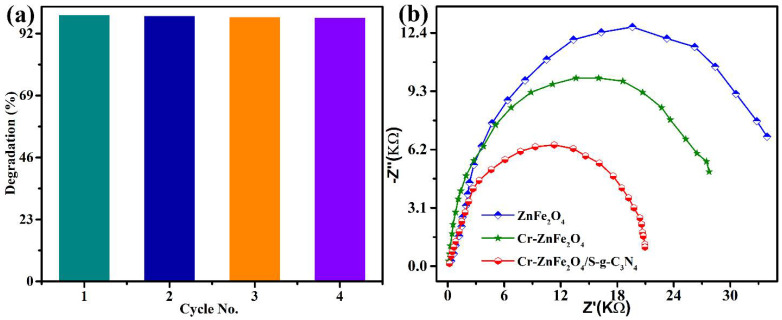
(**a**) Cyclic stability of the ZFG-50 NCs photocatalysts through the fourth cycle and (**b**) EIS Nyquist plots of ZnFe_2_O_4_, Cr-ZnFe_2_O_4_, and ZFG-50.

The photocatalyst’s durability during repeated photocatalytic activity is crucial for its practical uses. The ZFG-50 catalysts were recycled in five tests, and the material’s catalytic activity was tracked. In the recycling research, the ZFG-50 kept up its dye degradation rate. The composite’s dye degradation efficiency did not significantly decrease. According to the findings, even after the fourth cycle, effective dye degradation remained at over 95% (Figure 7a). The ZFG-50 catalysts might thus function as trustworthy, effective, and reusable photocatalytic materials. The ZFG-50 NCs’ crystal phase structure did not change significantly before or after the organic pollutants recycling experiments, according to the results of the XRD stability study, demonstrating chemical structural resilience (Figure S6). EIS in the dark was used to calculate the heterointerface charge transfer rate at the electrode–electrolyte junction. With a smaller arc radius and lower electron transport barrier, interfacial photoinduced charge transfer and departure efficiency is often faster. The heterointerface contact of the ZFG-50 may considerably help electron transmission, boosting electron consumption and enhancing photocatalytic performance, as shown by Figure 7b, which demonstrates that the ZFG-50 sample had the lowest charge-transmission resistance of all the produced samples. According to the experimental results, a ZFG-50 heterojunction may significantly improve light-collecting efficiency, effective separation of photogenerated e^−^ and h^+^ couples, and heterointerface electron transmission.

### 3.5. Photocatalytic Degradation Mechanism

**Figure 8:**
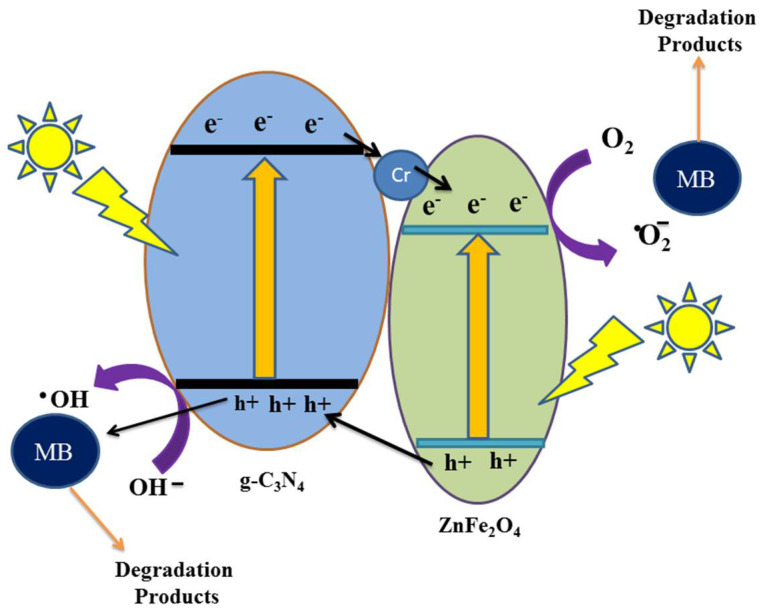
A schematic MB sunlight catalytic degradation mechanism over the ZFG NCs.

In the photocatalytic degradation mechanism as purposed in the schematic sketch (Figure 8), the enhanced degradation of methylene blue by photocatalysts may be ascribed due to the generation of e^−^/h^+^ pairs. EPR spectra of ZFG-50 NCs were explored to further corroborate the validation of functional species ·O_2_**^−^** and ·OH in the photodegradation mechanism (Figure S7a,b). When solar light is irradiated on ZFG, both Cr-ZnFe_2_O_4_ and S-g-C_3_N_4_ are energized and e^−^/h^+^ pairs are generated on their conduction band (CB) and valence band (VB), respectively [53]. Based on the CB/VB edge potentials, the photo-induced electrons can be easily migrated from the conduction band (CB) of Cr-ZnFe_2_O_4_ to the CB of S-g-C_3_N_4_ since the CB of Cr-ZnFe_2_O_4_ is lower than that of S-g-C_3_N_4_. At the same time, the holes generated in the VB of S-g-C_3_N_4_ could migrate to Cr-ZnFe_2_O_4_ [23]. The Cr atoms not only decrease the Eg value but also act as facilitators to transport e^−^ from S-g-C_3_N_4_ to ZnFe_2_O_4_ in the hybrid composite. Thus, doping could considerably reduce the possibility of photogenerated charge recombination by improving the separation of photogenerated e^−^/h^+^ pairs. The generated e^−^ & h^+^ reacts with the water and oxygen molecules absorbed on the surface of the photocatalyst and produce radicals (·OH and ·O^−2^) [8]. These radicals are utilized to break down MB by transforming it into low molecular weight intermediates, which are then changed into H_2_O, CO_2_, and inorganic ions via an oxidative mechanism. Equations (1)–(7) show the reductive and oxidative reactions involved in the photo-degradation of MB by ZFG NC.

(1) $$Cr-ZnFe_{2}\frac{O_{4}}{Sg-C_{3}N_{4}}+h\upsilon \to Cr-ZnFe_{2}\frac{O_{4}}{Sg-C_{3}N_{4}}\;(e^{-}/h^{+})$$

(2) $$h^{+}+H_{2}O\to H^{+}+\cdot OH$$

(3) $$2h^{+}+2H_{2}O\to 2H^{+}+H_{2}O_{2}$$

(4) $$H_{2}O_{2}\to 2\cdot OH$$

(5) $$2e^{-}+O₂\to \cdot O_{2}^{-}$$

(6) ·OH∕·O₂¯+MB→Degraded Products

(7) $$h^{+}+MB\to Degraded\;Products$$

### 3.6. Antibacterial Study

Both Gram-positive and Gram-negative bacteria were used to examine the antibacterial properties of ZnFe_2_O_4_, Cr-ZnFe_2_O4, and ZFG-50 NCs. Using the standard agar diffusion techniques, the antibacterial activity was carried out. *Staphylococcus aureus, Bacillus subtilis, Escherichia coli,* and *Streptococcus salivarius* were the four different bacterial strains used in the antibacterial tests. The Petri plates were taken out after the incubation period and placed under a laminar flow hood. Measurements and records of the zones of inhibition are provided in Table 4 for each sample, including the positive and negative controls. The zones of inhibition for each of the four bacterial strains against each of the four nanomaterials were measured and reported using the same method.

**Figure 9:**
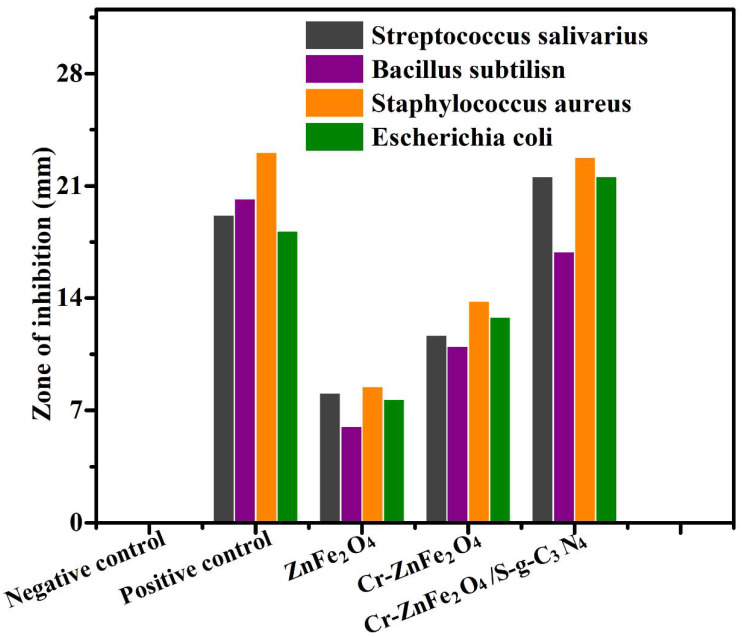
Zones of inhibition of the ZnFe_2_O_4_, Cr-ZnFe_2_O_4_, and ZFG-50 against the employed bacterial strains.

When exposed to the nanomaterials ZnFe_2_O_4_, Cr-ZnFe_2_O_4_, and ZFG-50 NCs, it was found that all four bacterial strains exhibited a zone of inhibition. While ZnO had the lowest bacterial inhibition zones, ZFG-50 NCs had the greatest. The increased surface area that the 7 percent Cr- ZnFe_2_O_4_ NPs allowed for surface contact NCs with bacterial membranes and the increased ROS generation brought on by the narrowing of the ZnFe_2_O_4_ bandgap may have contributed to the maximum antibacterial activity of the ZFG-50 NCs. All generated samples were examined for zones of inhibition against the four bacterial strains shown in Figure 9 and Table 4 below. The ternary composite has more antibacterial activity than the other synthetic nanomaterials, as seen in the bar graph below.

## 4. Conclusions

In conclusion, we have developed ZnFe_2_O_4_, Cr-ZnFe_2_O_4_ nanoparticles and a series of ZFG-50nanocomposites using a straightforward hydrothermal technique. The assembly and purity of samples were examined using XRD, EDX, and FTIR methods. ZnFe_2_O_4_, Cr-ZnFe_2_O_4_, and ZFG were used to degrade MB at ambient temperature. In a comparison photocatalytic investigation of the synthesized samples against MB, the ZFG-50 was found to have very high catalytic efficiency. A rate constant for the dye reduction reaction was discovered to be pseudo-first order both for NPs and NCs. Moreover, ternary composite ZFG-50 possesses significantly higher antibacterial activity compared to the other synthetic nanomaterials. Thus, ZFG-50 heterojunction is a promising candidate and has potential applications in the purification and disinfection of water by photocatalytic degradation of organic contaminants.

## Figures and Tables

**Table 1:** Composition of the synthesized Cr-ZnFe_2_O_4_/S-g-C_3_N_4_composites.

Sr. No.	Cr-ZnFe_2_O_4_ (wt. %)	S-g-C_3_N_4_ (wt. %)	7% Cr-ZnFe_2_O_4_/S-g-C_3_N_4_	Nanocomposites Code
1	-	100	S-g-C_3_N_4_	SG
2	100	-	ZnFe_2_O_4_	ZF
3	50	10	7% Cr-ZnFe_2_O_4_/10S-g-C_3_N_4_	ZFG10
4	50	30	7% Cr-ZnFe_2_O_4_/30S-g-C_3_N_4_	ZFG30
5	50	50	7% Cr-ZnFe_2_O_4_/50S-g-C_3_N_4_	ZFG50
6	50	60	7% Cr-ZnFe_2_O_4_/60S-g-C_3_N_4_	ZFG60
7	50	70	7% Cr-ZnFe_2_O_4_/70S-g-C_3_N_4_	ZFG70

**Table 2:** The rate constant (k) values of the ZFG nanocomposites.

Sr. No.	Nanocomposites	S-g-C_3_N_4_ (wt. %)	k (min^−1^)	Nanocomposites Code
1	S-g-C_3_N_4_	100	0.0021	SG
2	ZnFe_2_O_4_	-	0.0024	ZF
3	7% Cr-ZnFe_2_O_4_/10S-g-C_3_N_4_	10	0.0028	ZFG10
4	7% Cr-ZnFe_2_O_4_/30S-g-C_3_N_4_	30	0.0034	ZFG30
5	7% Cr-ZnFe_2_O_4_/50S-g-C_3_N_4_	50	0.0058	ZFG50
6	7% Cr-ZnFe_2_O_4_/60S-g-C_3_N_4_	60	0.0051	ZFG60
7	7% Cr-ZnFe_2_O_4_/70S-g-C_3_N_4_	70	0.0047	ZFG70

**Table 3:** Comparison of the ZFG-50 NCs’ photocatalytic effectiveness with some earlier research.

Scheme	Photocatalyst	Contaminant	Light Source	Radiation Time (min.)	Degradation %	Ref
1	ZnNd_x_Fe_2−x_O_4_	Rhodamine B	Xe lamp	180	98	[45]
2	N-ZnO/g-C_3_N_4_	MB	Xe lamp	90	100	[46]
3	Mn-ZnO/CSAC	BG	Solar	120	97.47	[47]
4	ZnFe_2_O_4_	Toluene	Xe lamp	300	57.2	[48]
5	ZnO/ZnFe_2_O_4_			100	98	[49]
5	Pt-BiFeO_3_	MG	Solar	240	96	[50]
7	g-C_3_N_4_/BiOI	RhB	Visible	120	99	[51]
8	ZnFe_2_O_4_@ZnO	MO	Visible	240	99	[52]
9	ZFG-50	MB	Solar	90	100	Present Work

**Table 4:** Bactericidal proficiency of ZnFe_2_O_4_, Cr-ZnFe_2_O_4_, and ZFG-50 NCs.

Antimicrobial Agent	*Escherichia Coli* (mm)	*Bacillus Subtilis* (mm)	*Streptococcus Salivarius* (mm)	*Staphylococcus Aureus* (mm)
Negative control	0	0	0	0
Positive control	18.2	20.2	23.1	19.2
ZnFe_2_O_4_	7.7	6	8.5	8.1
Cr-ZnFe_2_O_4_	12.8	11	13.8	11.7
ZFG-50	21.6	16.9	22.8	21.6

## Data Availability

The datasets generated during and/or analyzed during the current study are available from the corresponding author upon reasonable request.

## Supplementary Materials

The following supporting information can be downloaded at: https://www.mdpi.com/article/10.3390/molecules27196330/s1, Figure S1: High-resolution XPS spectra of Cr-ZnFe_2_O_4_/S-g-C_3_N_4_ NCs; (a) Zn 2p, (b) O 1s, (c) Fe 2p, (d) Cr 2p, (e) N 1s and (f) C 1s; Figure S2: (a) UV-vi absorption ranges and (b) Tauc’s plots of ZnFe_2_O_4_, S-g-C_3_N_4_, and Cr-ZnFe_2_O_4_/S-g-C_3_N_4_ NCs; Figure S3: The BET surface area isotherms estimated from N2 adsorption-desorption of ZnFe_2_O_4_, Cr-ZnFe_2_O_4_, S-g-C_3_N_4_, and Cr-ZnFe_2_O_4_/S-g-C_3_N_4_ NCs; Figure S4: Photodegradation of MB by Cr-ZnFe_2_O_4_ NPs after 150 minutes of sunlight irradiation (Degradation contours); Figure S5: Photodegradation of MB by Cr-ZnFe_2_O_4_/S-g-C_3_N_4_ NCs after 90 minutes of sunlight irradiation (Degradation contours); Figure S6: Structural stability of Cr-ZnFe_2_O_4_/S-g-C_3_N_4_ NCs identified by XRD patterns recorded before the first cycle and after the four-recycling test; Figure S7: ESR spectra of Cr-ZnFe_2_O_4_/S-g-C_3_N_4_ NCs: (c) in aqueous suspension for DMPO-•OH and (d) in methanol suspension for DMPO-•O_2_- under visible light radiance.

## Sample Availability

Samples of the compounds are not available from the authors.

## References

[1] Zhou Y., Jiao W., Xie Y., He F., Ling Y., Yang Q., Zhao J., Ye H., Hou Y. (2022). Enhanced photocatalytic CO_2_-reduction activity to form CO and CH_4_ on S-scheme heterostructured ZnFe_2_O_4_/Bi_2_MoO_6_ photocatalyst. J. Colloid Interface Sci..

[2] Sharma S., Dutta V., Raizada P., Hosseini-Bandegharaei A., Thakur V., Nguyen V.-H., VanLe Q., Singh P. (2021). An overview of heterojunctioned ZnFe_2_O_4_ photocatalyst for enhanced oxidative water purification. J. Environ. Chem. Eng..

[3] Yao Y., Cai Y., Lu F., Qin J., Wei F., Xu C., Wang S. (2014). Magnetic ZnFe_2_O_4_–C_3_N_4_ hybrid for photocatalytic degradation of aqueous organic pollutants by visible light. Ind. Eng. Chem. Res..

[4] Kuang M., Zhang J., Wang W., Chen J., Liu R., Xie S., Wang J., Ji Z. (2019). Synthesis of octahedral-like ZnO/ZnFe_2_O_4_ heterojunction photocatalysts with superior photocatalytic activity. Solid State Sci..

[5] Liang P.-L., Yuan L.-Y., Deng H., Wang X.-C., Wang L., Li Z.-J., Luo S.-Z., Shi W.-Q. (2020). Photocatalytic reduction of uranium (VI) by magnetic ZnFe_2_O_4_ under visible light. Appl. Catal. B Environ..

[6] Riaz K., Nadeem S., Chrouda A., Iqbal S., Mohyuddin A., Hassan S.U., Javed M., BaQais A., Tamam N., Aroosh K. (2022). Coupling of Se-ZnFe_2_O_4_ with rGO for spatially charged separated nanocomposites as an efficient photocatalyst for degradation of organic pollutants in natural sunlight. Colloids Surf. A Physicochem. Eng. Asp..

[7] Dai Z., Zhen Y., Sun Y., Li L., Ding D. (2021). ZnFe_2_O_4_/g-C_3_N_4_ S-scheme photocatalyst with enhanced adsorption and photocatalytic activity for uranium (VI) removal. Chem. Eng. J..

[8] Wu Y., Wang Y., Di A., Yang X., Chen G. (2018). Enhanced photocatalytic performance of hierarchical ZnFe_2_O_4_/g-C_3_N_4_ heterojunction composite microspheres. Catal. Lett..

[9] Pan Z.W., Wang R., Li J.N., Iqbal S., Liu W., Zhou K.B. (2018). Fe_2_P nanoparticles as highly efficient freestanding co-catalyst for photocatalytic hydrogen evolution. Int. J. Hydrog. Energy.

[10] Iqbal S., Bahadur A., Anwer S., Ali S., Irfan R.M., Li H., Shoaib M., Raheel M., Anjum T.A., Zulqarnain M.J.C. (2020). Effect of temperature and reaction time on the morphology of L-cysteine surface capped chalcocite (Cu_2_S) snowflakes dendrites nanoleaves and photodegradation study of methyl orange dye under visible light. Colloids Surf. A Physicochem. Eng. Asp..

[11] Palanivel B., Maiyalagan T., Jayarman V., Ayyappan C., Alagiri M. (2019). Rational design of ZnFe_2_O_4_/g-C_3_N_4_ nanocomposite for enhanced photo-Fenton reaction and supercapacitor performance. Appl. Surf. Sci..

[12] Kang S.G., Choe T.H., Ryom C.U., Ri M.C. (2021). Research on synthesis and photocatalytic activity of ZnFe_2_O_4_/Ag/g-C_3_N_4_ nanosheets composites. Compos. Interfaces.

[13] Hunge Y.M., Uchida A., Tominaga Y., Fujii Y., Yadav A.A., Kang S.-W., Suzuki N., Shitanda I., Kondo T., Itagaki M. (2021). Visible light-assisted photocatalysis using spherical-shaped bivo4 photocatalyst. Catalysts.

[14] Wang K., Li Q., Liu B., Cheng B., Ho W., Yu J. (2015). Sulfur-doped g-C_3_N_4_ with enhanced photocatalytic CO_2_-reduction performance. Appl. Catal. B Environ..

[15] Chen Y., Su F., Xie H., Wang R., Ding C., Huang J., Xu Y., Ye L. (2021). One-step construction of S-scheme heterojunctions of N-doped MoS_2_ and S-doped g-C_3_N_4_ for enhanced photocatalytic hydrogen evolution. Chem. Eng. J..

[16] Hong J., Xia X., Wang Y., Xu R. (2012). Mesoporous carbon nitride with in situ sulfur doping for enhanced photocatalytic hydrogen evolution from water under visible light. J. Mater. Chem..

[17] Fan Q., Liu J., Yu Y., Zuo S., Li B. (2017). A simple fabrication for sulfur doped graphitic carbon nitride porous rods with excellent photocatalytic activity degrading RhB dye. Appl. Surf. Sci..

[18] Jiang L., Yuan X., Pan Y., Liang J., Zeng G., Wu Z., Wang H. (2017). Doping of graphitic carbon nitride for photocatalysis: A reveiw. Appl. Catal. B Environ..

[19] Rostami M., Nayebossadr S., Mozaffari S., Sobhani-Nasab A., Rahimi-Nasrabadi M., Fasihi-Ramandi M., Ganjali M.R., Bardajee G.R., Badiei A. (2021). Heterojunction of N/B/RGO and g-C_3_N_4_ anchored magnetic ZnFe_2_O_4_@ ZnO for promoting UV/Vis-induced photo-catalysis and in vitro toxicity studies. Environ. Sci. Pollut. Res..

[20] Renukadevi S., Jeyakumari A.P. (2020). Rational design of ZnFe_2_O_4_/g-C_3_N_4_ heterostructures composites for high efficient visible-light photocatalysis for degradation of aqueous organic pollutants. Inorg. Chem. Commun..

[21] Zhang S., Rong X., Sun T., Gao P., Liu J., Qiu X., Zhou X., Wu Z. (2022). Enhancement of N_2_ adsorption by Z-scheme porous g-C_3_N_4_/ZnFe_2_O_4_ composite material for high-efficient photocatalytic nitrogen fixation. J. Porous Mater..

[22] Muhammad Irfan R., Hussain Tahir M., Maqsood M., Lin Y., Bashir T., Iqbal S., Zhao J., Gao L., Haroon M. (2020). CoSe as Non-Noble-Metal Cocatalyst Integrated with Heterojunction Photosensitizer for Inexpensive H_2_ Production under Visible Light. J. Catal..

[23] Shi Y., Li L., Xu Z., Sun H., Amin S., Guo F., Shi W., Li Y. (2022). Engineering of 2D/3D architectures type II heterojunction with high-crystalline g-C_3_N_4_ nanosheets on yolk-shell ZnFe_2_O_4_ for enhanced photocatalytic tetracycline degradation. Mater. Res. Bull..

[24] Tang H., Li R., Fan X., Xu Y., Lin H., Zhang H. (2022). A novel S-scheme heterojunction in spent battery-derived ZnFe_2_O_4_/g-C_3_N_4_ photocatalyst for enhancing peroxymonosulfate activation and visible light degradation of organic pollutant. J. Environ. Chem. Eng..

[25] Patil S., Bhojya Naik H., Nagaraju G., Viswanath R., Rashmi S. (2017). Synthesis of visible light active Gd^3+^-substituted ZnFe_2_O_4_ nanoparticles for photocatalytic and antibacterial activities. Eur. Phys. J. Plus.

[26] Ajithkumar P., Mohana S., Sumathi S. (2020). Synthesis, characterization, optical and photocatalytic activity of yttrium and copper co-doped zinc ferrite under visible light. J. Mater. Sci. Mater. Electron..

[27] Belakehal R., Atacan K., Güy N., Megriche A., Özacar M. (2022). Fabrication of heterostructured CdS/g-C_3_N_4_/ZnFe_2_O_4_ nanocomposite synthesized through ultrasonic-assisted method for efficient photocatalytic hydrogen production. Appl. Surf. Sci..

[28] Rong X., Liu S., Xie M., Liu Z., Wu Z., Zhou X., Qiu X., Wei J. (2020). N_2_ photofixation by Z-scheme single-layer g-C_3_N_4_/ZnFe_2_O_4_ for cleaner ammonia production. Mater. Res. Bull..

[29] Yang N., Hu P., Chen C., Wang Y., Pan L. (2019). Ternary Composite of g-C_3_N_4_/ZnFe_2_O_4_/Fe_2_O_3_: Hydrothermal Synthesis and Enhanced Photocatalytic Performance. ChemistrySelect.

[30] Iqbal S., Amjad A., Javed M., Alfakeer M., Mushtaq M., Rabea S., Elkaeed E.B., Pashameah R.A., Alzahrani E., Farouk A.-E.J.F.i.C. (2022). Boosted Spatial Charge Carrier Separation of Binary ZnFe_2_O_4_/Sg-C_3_N_4_ Heterojunction for Visible-light-driven Photocatalytic activity and Antimicrobial Performance. Front. Chem..

[31] Iqbal S., Javed M., Hassan S.S., Nadeem S., Akbar A., Alotaibi M.T., Alzhrani R.M., Awwad N.S., Ibrahium H.A., Mohyuddin A. (2022). Binary Co@ZF/S@GCN S-scheme heterojunction enriching spatial charge carrier separation for efficient removal of organic pollutants under sunlight irradiation. Colloids Surf. A Physicochem. Eng. Asp..

[32] Askari M.B., Salarizadeh P., Seifi M., Di Bartolomeo A. (2021). ZnFe_2_O_4_ nanorods on reduced graphene oxide as advanced supercapacitor electrodes. J. Alloy. Compd..

[33] Kalisamy P., Lallimathi M., Suryamathi M., Palanivel B., Venkatachalam M. (2020). ZnO-embedded S-doped gC_3_N_4_ heterojunction: Mediator-free Z-scheme mechanism for enhanced charge separation and photocatalytic degradation. RSC Adv..

[34] Yadav A., Kang S.-W., Hunge Y. (2021). Photocatalytic degradation of Rhodamine B using graphitic carbon nitride photocatalyst. J. Mater. Sci. Mater. Electron..

[35] Powar R.R., Phadtare V.D., Parale V.G., Pathak S., Piste P.B., Zambare D.N. (2019). Structural and magnetic properties of Cr-Zn nanoferrites synthesized by chemical Co-precipitation method. J. Korean Ceram. Soc..

[36] Hoque S.M., Hossain M.S., Choudhury S., Akhter S., Hyder F. (2016). Synthesis and characterization of ZnFe_2_O_4_ nanoparticles and its biomedical applications. Mater. Lett..

[37] Zhang W., Shen Y., Zhang J., Bi H., Zhao S., Zhou P., Han C., Wei D., Cheng N. (2019). Low-temperature H_2_S sensing performance of Cu-doped ZnFe_2_O_4_ nanoparticles with spinel structure. Appl. Surf. Sci..

[38] Kuai S., Zhang Z., Nan Z. (2013). Synthesis of Ce^3+^ doped ZnFe_2_O_4_ self-assembled clusters and adsorption of chromium (VI). J. Hazard. Mater..

[39] Alwan R.M., Kadhim Q.A., Sahan K.M., Ali R.A., Mahdi R.J., Kassim N.A., Jassim A.N. (2015). Synthesis of zinc oxide nanoparticles via sol–gel route and their characterization. Nanosci. Nanotechnol..

[40] Yan S., Li Z., Zou Z. (2009). Photodegradation performance of g-C_3_N_4_ fabricated by directly heating melamine. Langmuir.

[41] Vinoth S., Subramani K., Ong W.-J., Sathish M., Pandikumar A. (2021). CoS2 engulfed ultra-thin S-doped g-C_3_N_4_ and its enhanced electrochemical performance in hybrid asymmetric supercapacitor. J. Colloid Interface Sci..

[42] Farouq R. (2022). Coupling Adsorption-Photocatalytic Degradation of Methylene Blue and Maxilon Red. J. Fluoresc..

[43] Qamar M.A., Shahid S., Javed M. (2020). Synthesis of dynamic g-C_3_N_4_/Fe@ZnO nanocomposites for environmental remediation applications. Ceram. Int..

[44] Sher M., Shahid S., Javed M. (2021). Synthesis of a novel ternary (g-C_3_N_4_ nanosheets loaded with Mo doped ZnOnanoparticles) nanocomposite for superior photocatalytic and antibacterial applications. J. Photochem. Photobiol. B Biol..

[45] Nguyen L.T., Nguyen H.T., Le T.H., Nguyen L.T., Nguyen H.Q., Pham T.T., Bui N.D., Tran N.T., Nguyen D.T.C., Lam T.V. (2021). Enhanced photocatalytic activity of spherical Nd^3+^ substituted ZnFe_2_O_4_ nanoparticles. Materials.

[46] Kumar S., Kumar A., Kumar A., Balaji R., Krishnan V. (2018). Highly efficient visible light active 2D-2D nanocomposites of N-ZnO-g-C_3_N_4_ for photocatalytic degradation of diverse industrial pollutants. ChemistrySelect.

[47] Nithya R., Ragupathy S., Sakthi D., Arun V., Kannadasan N. (2020). A study on Mn doped ZnO loaded on CSAC for the photocatalytic degradation of brilliant green dye. Chem. Phys. Lett..

[48] Li J., Li X., Yin Z., Wang X., Ma H., Wang L. (2019). Synergetic effect of facet junction and specific facet activation of ZnFe_2_O_4_ nanoparticles on photocatalytic activity improvement. ACS Appl. Mater. Interfaces.

[49] Falak P., Hassanzadeh-Tabrizi S., Saffar-Teluri A. (2017). Synthesis, characterization, and magnetic properties of ZnO-ZnFe_2_O_4_ nanoparticles with high photocatalytic activity. J. Magn. Magn. Mater..

[50] Jaffari Z.H., Lam S.-M., Sin J.-C., Zeng H., Mohamed A.R. (2020). Magnetically recoverable Pd-loaded BiFeO_3_ microcomposite with enhanced visible light photocatalytic performance for pollutant, bacterial and fungal elimination. Sep. Purif. Technol..

[51] Wang J.-C., Yao H.-C., Fan Z.-Y., Zhang L., Wang J.-S., Zang S.-Q., Li Z.-J. (2016). Indirect Z-scheme BiOI/g-C_3_N_4_ photocatalysts with enhanced photoreduction CO_2_ activity under visible light irradiation. ACS Appl. Mater. Interfaces.

[52] Kulkarni S.D., Kumbar S., Menon S.G., Choudhari K., Santhosh C. (2016). Magnetically separable core–shell ZnFe_2_O_4_@ZnO nanoparticles for visible light photodegradation of methyl orange. Mater. Res. Bull..

[53] Zhang H., Zhu C., Zhang G., Li M., Tang Q., Cao J. (2020). Palladium modified ZnFe_2_O_4_/g-C_3_N_4_ nanocomposite as an efficiently magnetic recycling photocatalyst. J. Solid State Chem..

