# Supplementary material for: Fabrication of Cr-ZnFe2O4/S-g-C3N4 Heterojunction Enriched Charge Separation for Sunlight Responsive Photocatalytic Performance and Antibacterial Study
Source: Molecules. 2022 Sep 26;27(19):6330. doi: 10.3390/molecules27196330 (PMC9571418; doi:10.3390/molecules27196330)
Supplement: Supplementary file 1 [file molecules-27-06330-s001.zip › molecules-1909024-supplementary.pdf]

Supporting Information

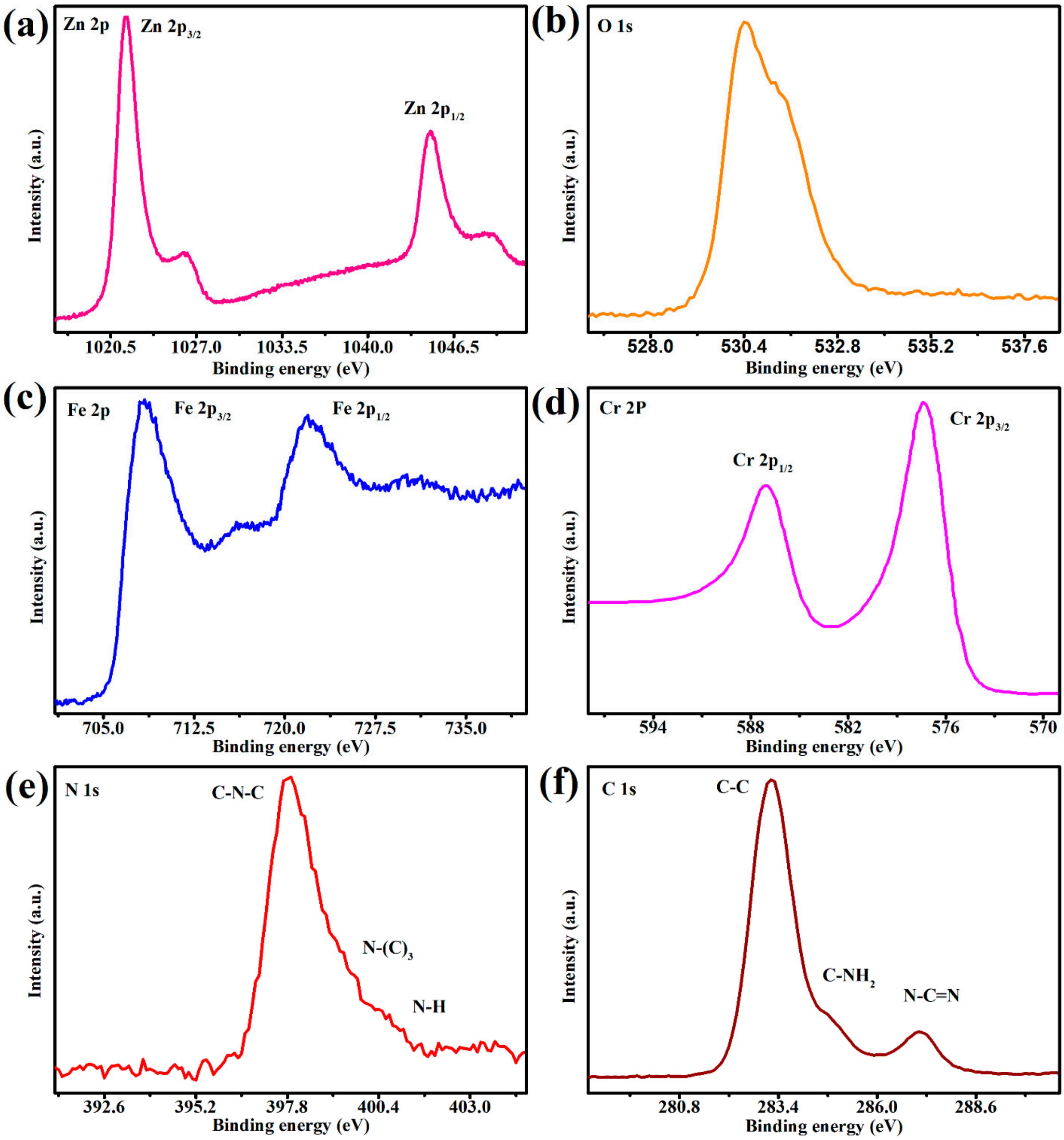

Figure S1. High-resolution XPS spectra of Cr-$ZnFe_2O_4$/S-g-C_3N_4 NCs; (a) Zn 2p, (b) O 1s, (c) Fe 2p, (d) Cr 2p, (e) N 1s and (f) C 1s.

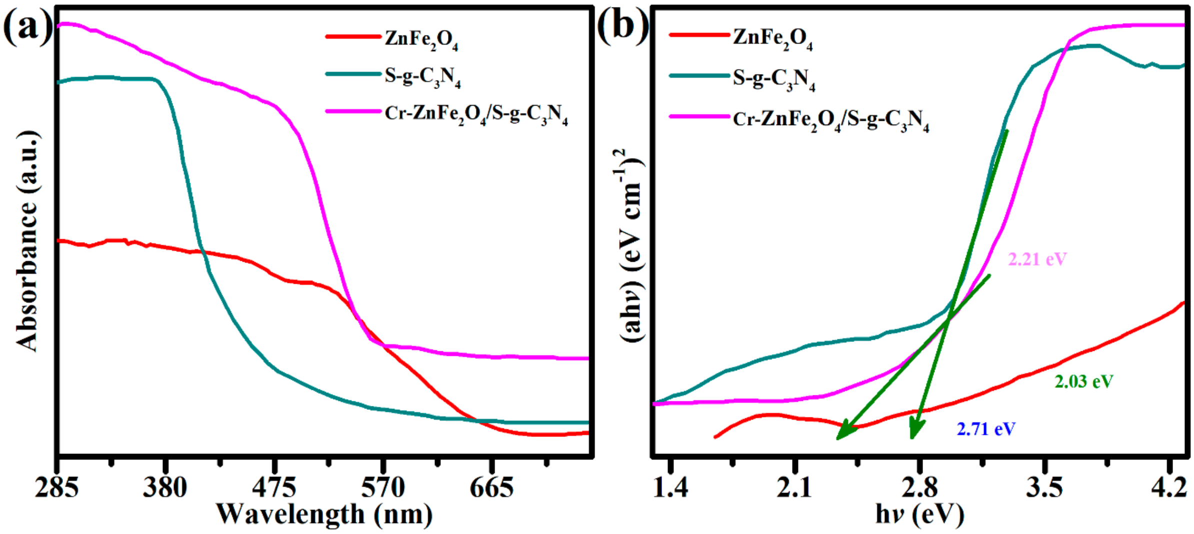

Figure. S2. (a) UV-vi absorption ranges and (b) Tauc's plots of $ZnFe_2O_4$, S-g-C_3N_4, and Cr-$ZnFe_2O_4$/S-g-C_3N_4 NCs.

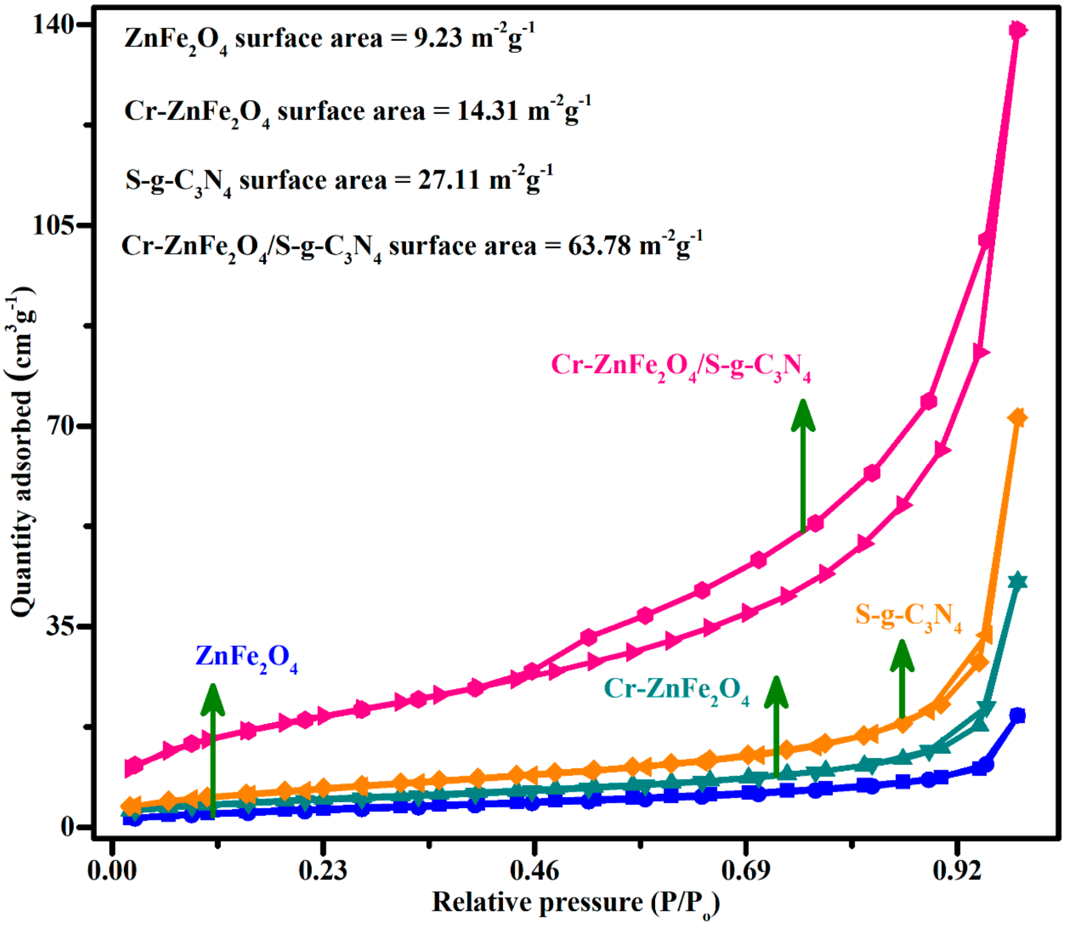

Figure. S3. The BET surface area isotherms estimated from N_2 adsorption-desorption of $ZnFe_2O_4$, Cr-$ZnFe_2O_4$, S-g-C_3N_4, and Cr-$ZnFe_2O_4$/S-g-C_3N_4 NCs.

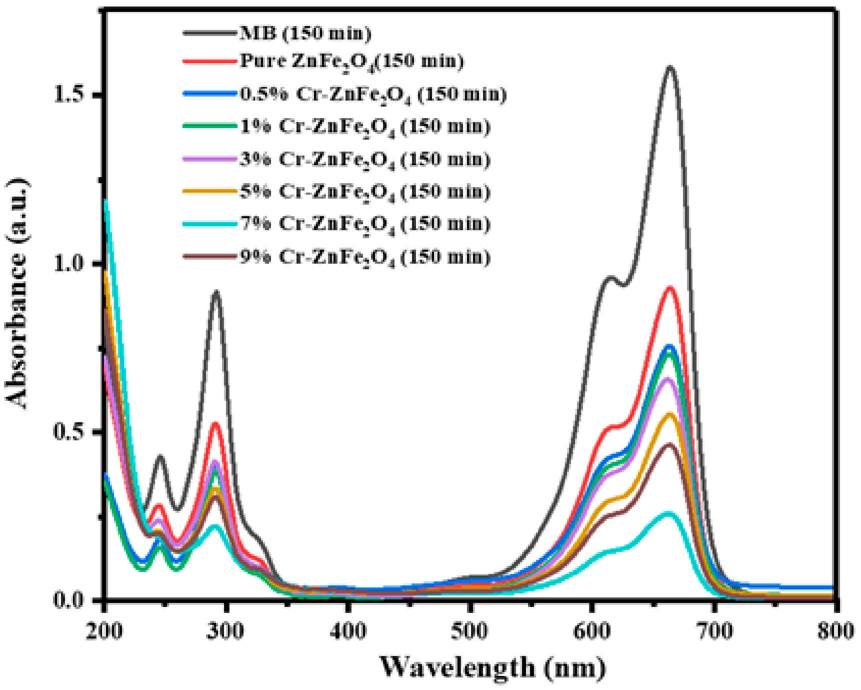

Figure. S4. Photodegradation of MB by Cr-$ZnFe_2O_4$ NPs after 150 minutes of sunlight irradiation (Degradation contours)

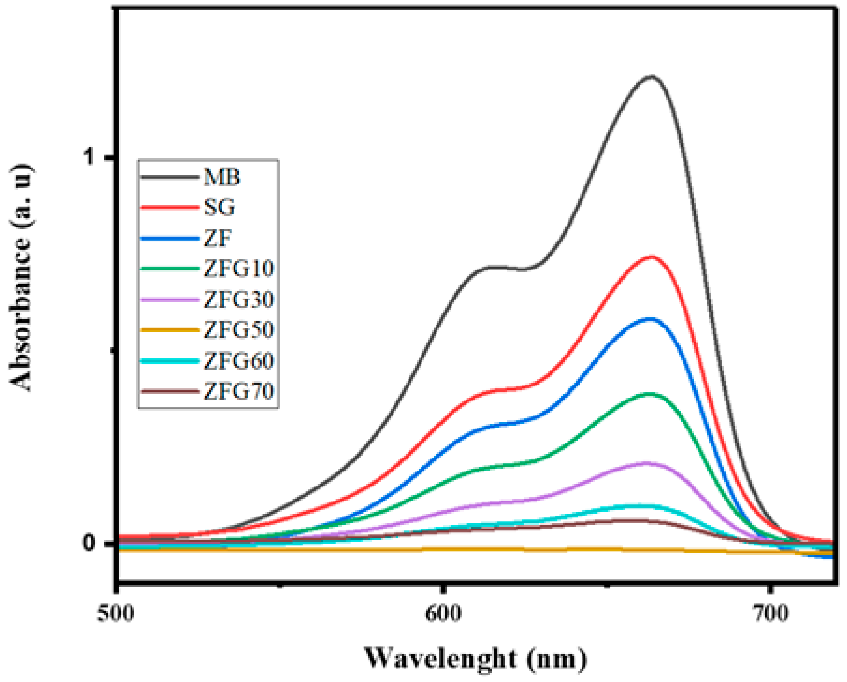

Figure. S5. Photodegradation of MB by Cr-$ZnFe_2O_4$/S-g-C_3N_4 NCs after 90 minutes of sunlight irradiation (Degradation contours).

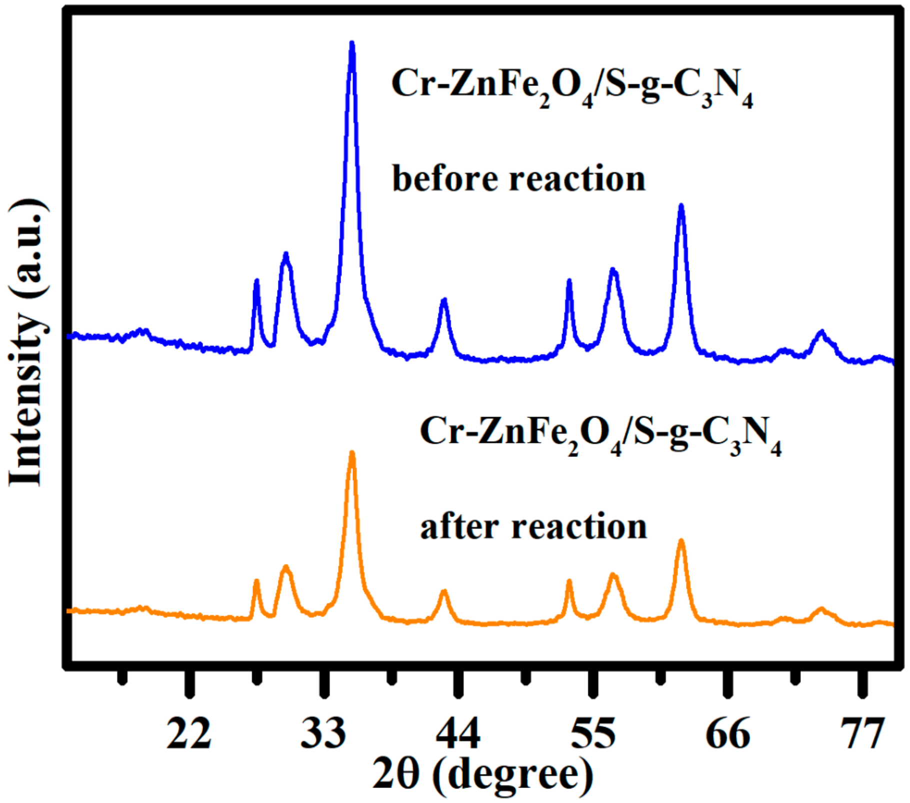

Figure. S6. Structural stability of Cr-$ZnFe_2O_4$/S-g-C_3N_4 NCs identified by XRD patterns recorded before the first cycle and after the four-recycling test.

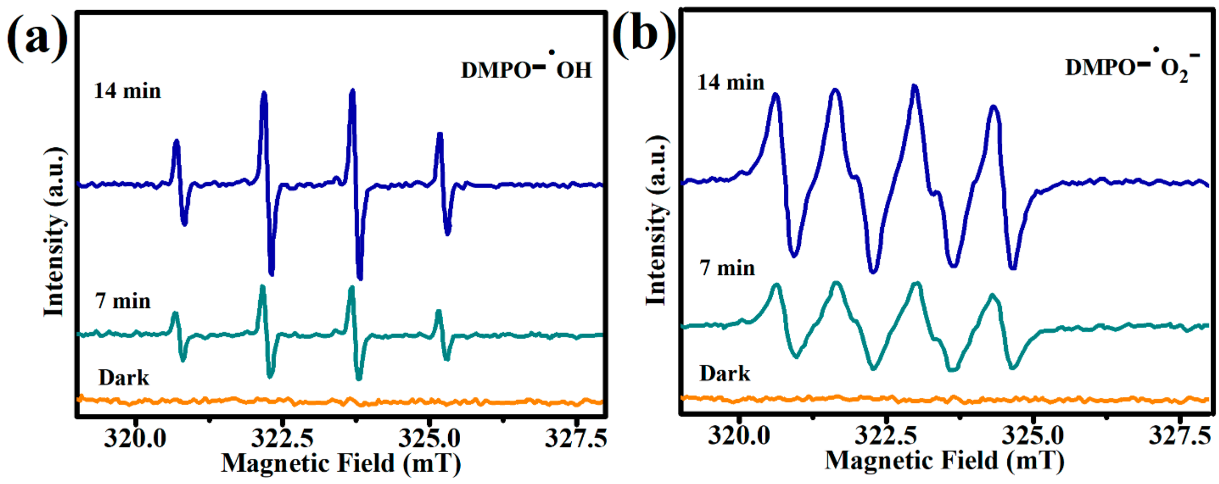

Figure. S7. ESR spectra of Cr-$ZnFe_2O_4$/S-g-C_3N_4 NCs: (c) in aqueous suspension for DMPO$^-$•OH and (d) in methanol suspension for DMPO$^-$•O_2^- under visible light radiance.
